# Outcomes of Diabetic Toe Amputation With Versus Without Metatarsal Head Resection for Single Ray Wet Gangrene: A Preliminary Study

**DOI:** 10.1002/jfa2.70052

**Published:** 2025-05-19

**Authors:** Kaissar Yammine, Mohammad Honeine, Joseph Mouawad, Ghadi Abou Orm, Youssef Jamaleddine, Chahine Assi

**Affiliations:** ^1^ Department of Orthopedic Surgery Lebanese American University Medical Center‐Rizk Hospital School of Medicine Lebanese American University Beirut Lebanon; ^2^ Diabetic Foot Clinic Lebanese American University Medical Center‐Rizk Hospital Beirut Lebanon; ^3^ Center for Evidence‐Based Anatomy, Sport & Orthopedics Research Beirut Lebanon

**Keywords:** diabetic foot infection, diabetic foot ulcer, osteomyelitis, toe amputation, wet gangrene

## Abstract

**Background:**

Diabetic wet gangrene of the toes is a serious condition that puts at risk the limb and life of patients. Two types of amputation are used when infection is around the metatarsophalangeal joint (MTPJ): complete toe disarticulation and toe amputation including metatarsal head resection. Because very few published papers analyzed the results of toe amputation for wet gangrene, our study aimed to evaluate the outcomes of both techniques.

**Methods:**

This is a retrospective comparative study of highly selective patients admitted for a single toe/ray diabetic wet gangrene that were treated with toe amputation through MTPJ (TA‐MTPJ) versus toe amputation with resection of the metatarsal head (TA‐MHR). Three primary outcomes were set for analysis: healing rate and the frequencies of infection recurrence and additional surgeries, including reamputations.

**Results:**

The sample included 31 cases: 12 cases (39%) with TA‐MTPJ and 19 cases (61%) with TA‐MHR. Outcomes of TA‐MTPJ versus TA‐MHR were as follows: (a) healing frequency 66.7% versus 58% (*p* = 0.6), (b) infection recurrence 50% versus 52.6% (*p* = 0.8), (c) osteomyelitis 41.6% versus 42.1% (*p* = 0.8), and (d) reamputation 33.3% versus 47.3% (*p* = 0.4).

**Conclusions:**

This study showed high complications after toe amputation for diabetic digital wet gangrene, with a trend for higher frequency of complications after TA‐MHR compared to TA‐MTPJ. For any type of amputation required for toe wet gangrene, it is likely that a more proximal level of index amputation is required.

## 1 Introduction

Patients with diabetic foot ulcers (DFU) or diabetic foot infections (DFI) are at high risk of lower extremity amputation (LEA) and premature death [1, 2]. Such complications could be more prevalent in the presence of severe infection or gangrene [3, 4]. In the context of advanced infection, aggressive treatment in the form of conservative surgery or amputation is the mainstay for infection control and limb salvage. Although conservative surgery could yield good to excellent outcomes for moderate to severe infections [5, 6], amputation is often needed when patients present with wet gangrene.

The forefoot is the most frequent location for diabetic ulcers and infections [7, 8]. Yet, few studies reported on the outcomes of surgical treatment in the toes [9, 10]. Toe wet gangrene is an urgent medical condition that requires prompt management, usually in the form of amputation.

Amputation levels in the forefoot comprise partial toe amputation, disarticulation at the level of the MTPJ, partial ray resection, total ray resection, and transmetatarsal amputations. Levels of amputation are decided depending on the level of proximal extent of infection. The most common types of toe amputation for wet gangrene reaching MTPJ are complete toe disarticulation and partial ray resection at the level of the distal metatarsal bone along with the involved toe. The decision on whether to use one technique over the other is difficult, usually depending on the proximal extent of bone and soft tissue infection. Because comparative results of both techniques are seldom reported, the aim of this early report is to document the indications and the outcomes after both types of toe amputation.

## 2 Methods

### 2.1 Study Design

This study included a continuous series of patients treated for forefoot wet gangrene. A retrospective comparative design was created to look for outcome differences between two groups: toe amputation through MTPJ (TA‐MTPJ) versus toe amputation with resection of the metatarsal head (TA‐MHR). The electronic charts of patients admitted between January 2019 and August 2022 were checked, and relevant data were extracted. Approval by the institutional review board was obtained prior to study conduction. Guidelines of the Joanna Briggs Institute checklist for cohort studies were followed throughout this study [11].

### 2.2 Criteria for Patient Inclusion and Exclusion

All charts of patients presented with a clinical diagnosis of forefoot wet gangrene due to infected diabetic foot ulcers (Stage 4 of Wagner's classification) were checked. The diagnosis was based on clinical examination, and patients were admitted for amputation surgery. Only those with a single toe/ray involvement were included. Peripheral artery evaluation was performed in all cases by the vascular team, and patients who underwent revascularization prior to the amputation were accepted for inclusion. Patients who had a partial toe amputation (proximal or distal interphalangeal disarticulation) where one or more phalangeal segments were kept were not included. Cases of dry necrosis of a toe due to PAD were excluded. Wet gangrene of the mid‐foot (or reaching the mid‐foot) and of the hindfoot was also excluded. All included patients were scheduled for urgent surgery within 24 h of admission. Because of the longstanding cooperation with our radiology department, all patients underwent MRI prior to surgery. Patients who received broad‐spectrum antibiotics prior to surgery were excluded. The minimum follow‐up period for inclusion was 12 months.

### 2.3 Surgical Indication and Group Comparison

The indication of the index amputation depended on two elements: the level of soft tissue involvement as clinically observed and/or the level of bone involvement based on MRI results.

The TA‐MTPJ group (disarticulation group) included cases in which the amputation was performed at the MTPJ due to gangrene distal to the MTPJ level, with at least 1 cm of healthy soft tissue. In this group, no MRI or peroperative signs of septic arthritis or metatarsal cartilage involvement were present. In this group, the most proximal accepted level of osteomyelitis for inclusion was the base of the first phalanx. The TA‐MHR group comprised those cases in which the metatarsal head was excised along with the toe amputation when (a) the necrosis was too close to the MTPJ (within 1 cm), (b) septic arthritis of the MTPJ or involvement of the metatarsal head was diagnosed prior to surgery by MRI, or (c) those infection conditions were suspected during the surgery. All specimens were sent for microbiology culture, and broad‐spectrum antibiotics were initiated in the operating room immediately after the procedure and deflation of the tourniquet, if any. Wounds were closed without tension in all patients. Antibiotherapy was later adapted to the found microorganism(s).

### 2.4 Outcome Definition

Three primary outcomes were set for analysis: healing rate and the frequencies of infection recurrence and additional surgeries, including reamputations. The healing rate reflects cases in which the primary index procedure with or without further surgical soft tissue debridement for superficial infection yielded no bone infection recurrence with complete wound healing. Infection recurrence was subcategorized into four subgroups: same ray bone infection, same ray soft tissue infection, adjacent ray bone infection, and adjacent ray soft tissue infection. Additional surgeries were also divided into five subgroups: surgical debridement, partial ray resection, total ray resection, transmetatarsal amputation, and below‐knee amputation (BKA). Mortality rate was set to be a secondary outcome.

### 2.5 Postoperative Protocol

All patients started broad‐spectrum antibiotics peroperatively after completion of the index amputation surgery and release of the tourniquet, when used. All specimens were sent for microbiology examination. Antibiotherapy was then adapted based on the found microorganism(s) and antibiograms.

### 2.6 Data Extraction and Analysis

Relevant demographic, risk factor, and outcome data were extracted and recorded into an Excel sheet. Continuous variables were expressed as means ± SD values and compared using the Student’s *t*‐test. Categorical variables were expressed as frequencies and compared using the chi‐squared test. The StatsDirect software (Cambridge, UK) was used for statistical analysis. A *p*‐value less than 0.05 was considered to be significant.

## 3 Results

### 3.1 Sample Characteristics

A total of 28 patients were included in this study. Gender distribution was as follows: 22 males (78.5%) and 5 females (21.5%). In total, 31 cases met the inclusion criteria. There were 25 cases on the right side and 6 cases on the left side. The disarticulation group included 12 cases (39%), and the TA‐MHR group comprised 19 cases (61%). The mean age of the whole sample was 72.3 ± 15.4 years. The mean ASA score was 2.8 ± 0.4. The mean follow‐up period was 30.3 ± 14.2 months. Table 1 summarizes the characteristics and risk factors of both groups of the sample.

**TABLE 1:** Characteristics of the sample.

	TA‐MTPJ	TA‐MHR	*p‐*value
Nb cases	12	19	—
Age (years)	75.4 ± 13.6	70.3 ± 16.6	0.2
ASA score	2.8 ± 0.4	2.7 ± 0.4	0.8
Follow‐up (months)	32.5 ± 16	29 ± 13.2	0.3
HTN	12 (100%)	16 (84.2%)	0.3
PAD	4 (33.3%)	8 (42%)	0.6
DL	6 (50%)	14 (73.7%)	0.2
CAD	7 (58.3%)	16 (84.2%)	0.1
CKD	7 (58.3%)	15 (79%)	0.2
Smoking	8 (66.7%)	16 (84.2%)	0.2
Hg	10.4 ± 3.9	10 ± 2.1	0.9
Creatinine	2.08 ± 1.2	1.8 ± 1.1	0.7

### 3.2 Location of Wet Gangrene and Osteomyelitis Occurrence

In the TA‐MTPJ group, most gangrene cases were located in the three most lateral toes, whereas in the TA‐MHR group, highest frequencies were noted in the hallux and the fourth toe. Table 2 shows details on gangrene's location. MRI signs were suggestive of osteomyelitis in 64% and 72% for the TA‐MTPJ and the TA‐MHR groups, respectively (*p* = 0.5).

**TABLE 2:** Location of gangrene.

Location	TA‐MTPJ	TA‐MHR
Hallux	1	6
Second toe	1	2
Third toe	3	3
Fourth toe	3	5
Fifth toe	4	3
Total	12	19

### 3.3 Healing Rate

In all cases, primary closure of the wounds was achieved. The healing rates after the index procedure with or without further soft tissue debridement were 66.7% and 58% for the TA‐MTPJ and the TA‐MHR groups, respectively (*p* = 0.6). All five cases treated with TA‐MHR where necrosis was too close to the MTPJ with bone infection distal to it healed with no complications.

### 3.4 Frequency of Infection Recurrence

Infection recurrence was 50% and 52.6% for the TA‐MTPJ and the TA‐MHR groups, respectively (*p* = 0.8). Most infection recurrences were in the form of osteomyelitis either in the same ray or any adjacent ray (41.6% vs. 42.1%, *p* = 0.8). No significant differences could be found between both groups in any subcategory. Table 3 recorded the details of infection recurrence.

**TABLE 3:** Outcome frequencies.

Outcomes	TA‐MTPJ^a^	TA‐MHR^a^	*p‐*value
Total number of cases	12	19	—
Infection recurrence	6 (50%)	10 (52.6%)	0.8
Same ray bone infection	3 (25%)	4 (21%)	0.8
Same ray soft tissue infection	1 (8.3%)	2 (10.5%)	0.8
Adjacent ray bone infection	2 (16.7%)	4 (21%)	0.7
Adjacent ray soft tissue infection	1 (8.3%)	0	0.9
Additional surgeries	6 (50%)	10 (52.6%)	0.8
Surgical soft tissue debridement	2 (16.7%)	1 (5.2%)	0.3
Partial ray resection	4 (33.3%)	5 (26.3%)	0.7
Total ray resection	0	2 (10.5%)	0.7
Transmetatarsal amputation	0	0	—
Below‐knee amputation	0	2 (10.5%)	0.7

^a^ *p*‐values did not reach significance for all outcomes.

### 3.5 Frequency of Additional Surgeries

Half of the cases in both groups required an additional surgery, mostly in the form of a reamputation. Reamputation was required in 33.3% and 47.3% of cases in the TA‐MTPJ and TA‐MHR groups, respectively (*p* = 0.4). Major amputations were recorded only in the TA‐MHR group with a frequency of 10.5%. No significant differences could be found between both groups in any other subcategory. Table 3 summarizes details of the additional surgeries.

**FIGURE 1:**
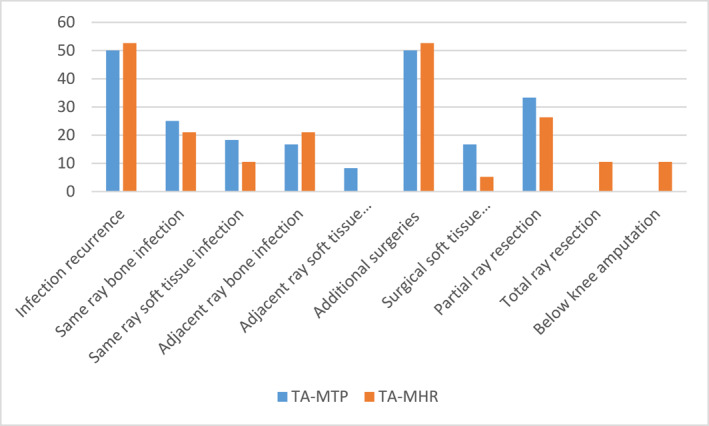
Complication frequency comparison.

Figure 1 compares complication frequencies of both procedures.

### 3.6 Mortality Rate

No death was recorded during the follow‐up period.

## 4 Discussion

This preliminary study highlights the seriousness of wet gangrenes occurring in patients with diabetic foot disease. Even when treated within 24 h, the frequency of complications is excessively high. With a radical surgical treatment such as amputation with or without superficial debridement, the rate of healing did not reach 70%. Infection recurred in half of the cases, mostly a bone infection at the initial or adjacent toe/ray site. A more proximal reamputation was needed for approximately one‐third (TA‐MTPJ group) and half of the cases (TA‐MHR group). Our findings show that the outcomes of both forms of amputation surgery are not optimal, and consequently, there is a need to appraise the current surgical indications.

Very few studies compared both techniques in terms of postoperative complications. Tenze et al. reported significantly better healing time for those who had a toe amputation, including MHR, compared to toe disarticulation, but with no difference in ulcer healing rate [12]. In contrast, Nehler et al. studied the intermediate outcomes of any form of forefoot sepsis diagnosed by physical examination and plain radiographs [13]. They concluded that disarticulation at any toe joint level is 2.7 times more likely to heal than those including the metatarsal head. Additionally, those authors reported that healing rates tend to decrease as greater bone resection occurred. Twenty‐five years later, our study showed the same trend when both groups are compared, despite the use of MRI information. The systematic use of MRI in our institution, unfortunately, did not reduce the reamputation rate. A probable cause for that could be the classical interpretation of bone signal on MRI. Signals on MRI tend to overestimate osteomyelitis diagnosis, the reason why the intramedullary edema signal is often interpreted as reactional. Our findings indicate that those signals might be due to an active infection rather than a reactional edema. Further studies are warranted to evaluate the concordance between MRI signs and bone infection on pathology examination.

Our results are similar to a published report by our team where high complications were found in cases after either any form of toe amputation distal to MTPJ or any form of ray resection surgery [10]. When treating severe diabetic toe infections (Grade 3 or 4 as per the International Working Group on the Diabetic Foot), osteomyelitis recurrence was found in approximately half of the cases and reamputation in approximately one‐third of the cases. Additionally, the abovementioned study reported significantly higher frequency of reamputation after ray resection compared to toe amputation. Our study showed a similar trend when more resection is needed, though the difference did not reach significance.

Our findings, along with those few that analyzed the outcomes of digital amputation in diabetic foot infection, demonstrated that current indications failed to locally control infection. For instance, the 1 cm of healthy tissue criterion could not prevent infection recurrence in 50% of cases of the MTPJ disarticulation group. It is of importance to note that when soft tissue infection was less than 1 cm of the MTPJ but with bone infection distal to this level, resecting the metatarsal head for tension‐free wound closure yielded complete healing in all 5 cases with no complications. Resecting far from the initial bone infection seems to produce better outcomes. Similarly to our previous statement [10], a more proximal level of amputation could be more efficient when dealing with severe diabetic toe infections. Based on the observed high frequencies of osteomyelitis recurrence and reamputation, we suggest a more radical approach than that proposed by current practice. When a wet gangrene presents distal to the MTPJ, the metatarsal head is to be excised. When the gangrene reaches the MTPJ or in case of suspicion infection involving the metatarsal head, a ray resection up to the proximal one‐third of the metatarsal is to be performed.

### 4.1 Limitations

We acknowledge that our study constitutes a preliminary comparative report on the outcomes of diabetic toe wet gangrene. The small sample size reflects the imposed multiple criteria for inclusion, such as the systematic use of MRI, single toe/ray involvement, exclusion of partial toe amputations, and a diagnosis of wet gangrene limited to patients with diabetic foot disease. The retrospective design of this study bears its own limitations when compared to a prospective one. Controlled trials with a larger sample size would be necessary to confirm or infirm our findings. However, we hope that this study will serve as a solid base for future research in evidence‐based diabetic foot surgery.

## 5 Conclusion

In the frail population of patients with diabetic foot complications, every effort should be made to reduce the medical burden and optimize our treatment. This is truer when dealing with wet gangrene where radical interventions such as amputation surgery are needed to control the spread of infection and enhance limb salvage. This study showed high complication rates after toe amputation for diabetic digital wet gangrene. The findings highlight the need for a more radical change in our current indications and recommend a more proximal level of amputation than is commonly practiced. Keeping further away from the presumed infection could reduce infection recurrence and limit the number of reamputations.

## Author Contributions

**Kaissar Yammine:** conceptualization, methodology, formal analysis, investigation, writing – original draft, supervision. **Mohammad Honeine:** formal analysis, writing – original draft. **Joseph Mouawad:** investigation, writing – original draft. **Ghadi Abou Orm:** formal analysis, writing – original draft. **Yousef Jamaleddine:** investigation, writing – original draft. **Chahine Assi:** writing – review and editing, resources, supervision.

## Ethics Statement

This study was approved by Lebanese American University's Research Ethics Board (LAUMCRH.KY2.25/Nov/2020).

## Conflicts of Interest

The authors declare no conflicts of interest.

## Data Availability

Data are available upon request from the authors. The data that support the findings of this study are available from the corresponding author upon reasonable request.
